# What Is a Cure?

**Published:** 1896-10-24

**Authors:** 


### What is a Cure?

In regard to some diseases this might seem a foolish question. When the patient is well he is "cured"—whether by nature or by art has nothing to do with the matter, he is "cured." But there are maladies in which the problem is much more complex, and in which it is by no means easy to say whether a second attack is due to an imperfect cure in the first instance, or is the result of a persistence of that constitutional peculiarity by virtue of which the disease has been able to inflict itself upon the [illegible]tient on each occasion.

In regard to cancer and its curability, this question [illegible] not only of great interest to the unfortunate sufferers, but is of the extremest importance to the surgeon who has to decide upon treatment, for his readiness to perform large operations hinges upon his confidence in being able by them to remove the whole of the disease. Unless a surgeon were able to offer to his patient at least a fair probability of a "cure," it is hardly to be imagined that he could persuade his patient to undergo, any more than he would be likely himself to undertake, such extensive operations as are now performed for the removal of cancer of the breast. Yet it is maintained by some that in all ordinary cases of this disease infection of bone marrow occurs within six months of the inception of the malady, and that when that event has taken place, "cure" is out of the question. Many thing arise in regard to this which it would be quite out of the question to attempt to discuss in a short article. It does, however, seem worth while to point out that the the dread of marrow infection has not prevented those who believe in it from practising extensive operations, even although they may do so with but little hope of a complete and radical cure, and we cannot but feel that behind the assertion that marrow infection takes place within six months of the inception of the cancerous processes, there lingers the idea, and in fact the hope, that this infection may be so slow in its development as really to approximate rather to what some might call a constitutional tendency than to the progressive and spreading disease which we are apt to associate with the word "infection."

Are we not then playing upon words? Even those who believe most strongly in the local origin of cancer, and in its cure by operation, admit that cases do occur in which a very late recurrence does take place, although, in view of the steady diminution of recurrences in cases which have safely tided over a few years, they have come to adopt Volkmann's rule, to assume that a patient who shows no disease for three years after an operation may be looked upon as cured, and to ascribe disease appearing locally at a later period to a fresh development, and not to a residue left by the old condition; while those who maintain that, after cancer has existed for a few months, cure is impossible, also admit that people may live for years without suffering from any influence with the health, even though they have a marrow infection which may ultimately cause trouble.

There is, however, a considerable practical difference between the disciples of the two schools. Both would operate, and both would agree that success depends largely on early and extensive removal of the disease; but the one would continue to urge extensive operations in the hope of cure in cases so advanced in regard to time that in the view of the other all hope of cure was past, and dangerous operative proceedings were unjustifiable. It is an anxious question, and one that can only be decided by counting cures and observing dates, and certainly of late years those who have operated boldly and freely even in advanced cases have had great reward; although still it may be said that there is no common ground as to "what is a cure?"

